# Single and double bosonic stimulation of THz emission in polaritonic systems

**DOI:** 10.1038/srep05444

**Published:** 2014-06-25

**Authors:** M. A. Kaliteevski, K. A. Ivanov, G. Pozina, A. J. Gallant

**Affiliations:** 1 St-Petersburg Academic University Khlopina 8/3, 194021, 194021, St-Petersburg, Russia; 2 Ioffe Physical-Technical Institute of Russian Academy of Science, Polytechnicheskaya 26, 194021, St-Petersburg, Russia; 3 Department of Physics, Chemistry and Biology (IFM), Linköping University, S-58183 Linköping, Sweden; 4 School of Engineering and Computing Sciences, Durham University, South Road, Durham, UK DH1 3LE

## Abstract

The influence of the surrounding cavity on the efficiency of different types of polaritonic emitters of THz radiation has been analysed. It is demonstrated that THz lasing threshold in realistic structures cannot be achieved without a THz cavity, due to destruction of polaritons via excitonic Mott transition. Even modest values of cavity quality factor (not exceeding 50) provide significant quantum efficiency.

The development of compact, cheap and reliable solid state sources of THz radiation remains one of the unresolved problem of modern technology due to a low rate (of the order of inverse milliseconds) of THz radiative transitions while the lifetime of the excited charge carriers is typically shorter, i.e. in picosecond range^1^,^2^,^3^,^4^,^5^,^6^,^7^. Recently, polaritonic emitters of THz radiation have been proposed where the THz radiative transition occurs into a polariton condensate state and therefore experiences bosonic stimulation^8^. Moreover, polariton condensation and lasing has been experimentally demonstrated for quantum microcavities based on GaAs^9^, CdTe^10^ and GaN^11^. Soon after the suggestion of the basic idea for polaritonic emitters^8^, several possible schemes of such devices have been proposed^12^,^13^,^14^,^15^. In standard lasers, radiative transitions occur between different quantum states of electrons, namely particles which are fermions. Thus, the occupancy numbers of the electronic states does not exceed unity. In this case, lasing threshold is achieved when the population of the optical photonic (bosonic) mode reaches a threshold value^16^. In polaritonic THz devices, however, the radiative THz transition can be stimulated via two different mechanisms: either when (i) the population of a polaritonic condensate state or (ii) a THz mode reaches threshold value. In later case, the rate of radiative transition, accompanied by a THz emission, significantly increases. This paper is aimed to compare the efficiency of the THz emission of polaritonic THz devices operating in the regime of single bosonic stimulation when stimulation is provided by formation of only polaritonic condensate, and in the regime of double bosonic stimulation when the populations of both polariton condensate and the THz mode are above the threshold.

## Results

**Figure 1:**
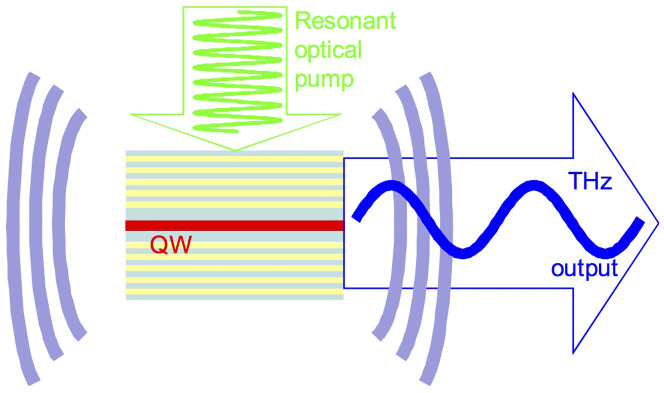
Schematic drawing of the structure for a polaritonic THz emitter: polaritonic microcavity with a quantum well is placed into the cavity with THz radiation.

In our consideration, the accumulation of THz photons has been provided by placing polaritonic emitter into an additional THz radiation cavity which is reasonable since high Q (~1500 at 0.3 THz) cavities in the THz region have been recently experimentally demonstrated using a Distributed Bragg Mirror approach^17^. For that, stacked layers of high resistivity silicon, with well-defined thicknesses and air gaps, have been constructed to flank, thus forming a cavity. We will consider here a structure for polaritonic THz emitter consisting of polaritonic microcavity with a quantum well placed into an additional cavity constructed for THz radiation as schematically shown in Fig. 1.

Confinement of the THz photons in the cavity leads to increasing photon lifetime *τ*, which become proportional to the cavity quality factor *Q*: *τ* = *τ*_0_*Q*, where *τ*_0_ is the lifetime of THz photons without the THz cavity. Further, the cavity presence affects emission process via Purcell effect^18^.

The characteristic probability *W*_0_ of the THz photon spontaneous emission in the free space due to radiative transitions between polaritonic levels is estimated using a Fermi golden rule, which gives: 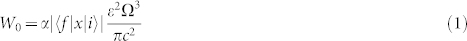
where α ≈ 1/137 is a fine structure constant, *ε* is a dielectric constant and Ω is a Rabi splitting of the polariton modes. Since the value of the matrix element 〈*f*|*x*|*i*〉 is usually a fraction of the Bohr radius *a_B_* (typical value of this quantity is about *a_B_/5*), the maximal value of the probability of the radiative THz transition in polaritonic emitter, *W_max_*, can be estimated as 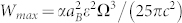. This gives an upper limit of the radiative THz transition rate. We have calculated *W_max_* presented in Table 1 for two types of microcavities, bulk and with embedded quantum well (QW), constructed on several semiconductor materials. Typical values of Rabi splitting 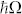, exciton Bohr radius *a*_B_, and dielectric constant *ε* are taken from Ref. (9,10,11, 19,20,21,22,23,24).

As aforementioned, the presence of the THz cavity will modify the emission probability via Purcell effect. Thus, in phenomenological approach assuming that probability of emission increases via Purcell effect, we can write: 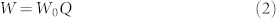
At the same time, the framework of common models for typical lasers, in the case when radiative broadening is much smaller than non-radiative broadening *γ*, the emission probability does not depend on the cavity Q-factor and is given by^16^

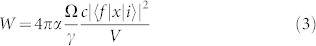
where *V* is a mode volume.

In the present study, we have considered three types of polaritonic THz emitters: (i) emitters based on mixing of the upper polariton state with the dark exciton^8^; (ii) polaritonic cascade lasers^12^,^13^; and (iii) emitters based on the transition between a dark 2*p*-exciton and a polariton condensate formed by an *s*-exciton^14^.

For each system we have analysed the population of polaritonic states and the THz mode using a set of steady-state Boltzmann equations, specific for each type of emitter, described in details in Methods. We calculate the occupancies of the THz mode, the polaritonic states and a quantum efficiency of the emitters as a function of the pumping rate and the THz cavity quality factor Q. For all considered systems we have used the same probability of the spontaneous emission *W*_0_ = 10^3^ s^−1^ and the same polariton decay time *τ*_0_ = 1 ps. The THz emitter based on mixing of the upper polariton state with the dark exciton THz emitter based on polaritonic cascade lasers (i.e. cases (i) and (ii), respectively) will be analysed using Eqs. 2 and 3. For the last type of the THz emitter (iii) the emission probability used was taken to *W* = 10^3^ s^−1^.

Polaritons are formed by coupled photons and excitons, which sets an upper limit for polariton concentration in the system. When distances between excitons become comparable to the Bohr radius *a_B_*, excitons start to screen each other and the exciton gas is replaced by the electron-hole plasma^25^, which corresponds to two-dimensional (2D) exciton density of ~10^12^ cm^−2^. Other estimates show even smaller 2D exciton Mott density, of the order of ~10^11^ cm^−2^ 9.

We note here that photonic disorder, which inevitably presents in microcavity structures, sets the upper limit for a characteristic size of polariton condensates. Experimentally observed polariton condensates have a typical size of 10 μm × 10 μm^11^,^26^, which, together with a characteristic exciton Bohr radius of the order of 10 nm or less, limits the maximal number of polaritons in the system to ~5 × 10^6^.

**Figure 2:**
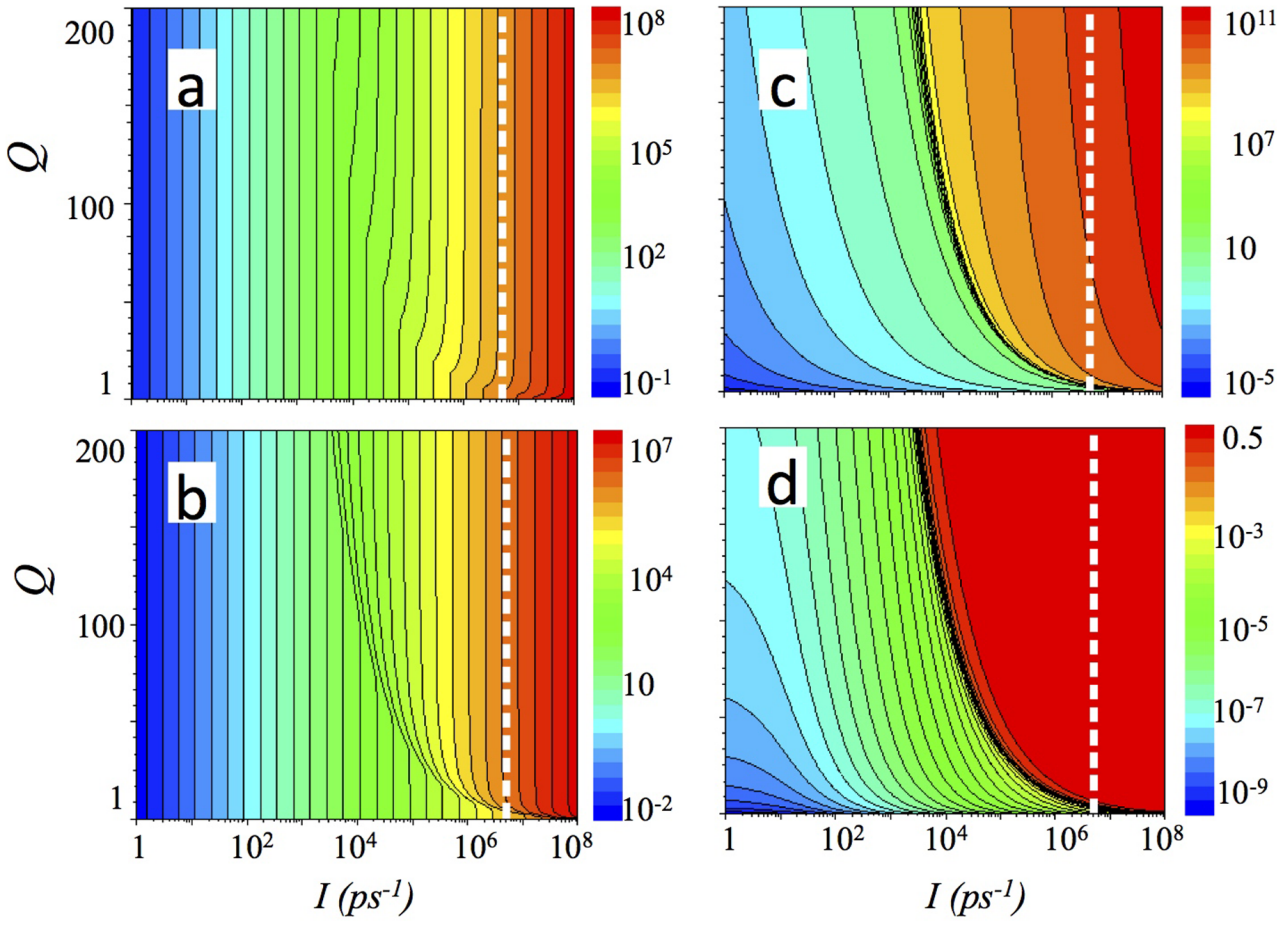
Populations (a) of the lower (L) and (b) of the upper (U) polaritonic levels, (c) of the THz mode T and (d) an external quantum efficiency as a function of pumping rate *I* and the quality factor of the THz cavity Q for polaritonic THz emitter based on mixing of the upper polariton state with the dark exciton. Calculations are done using Eq. 4–7. A modelling was performed using Eq. 2 for emission probability. The following parameters have been used: *τ_L_* = 1 ps, *τ_U_* = 1 ps, *τ_R_* = 100 ps, *τ_UR_* = 10 ps, *τ_LR_* = 10 ps. The dashed white isoline shows a pumping level corresponding to 5 × 10^6^ polaritons in the system.

Taking into account abovementioned issues, we have calculated populations for the polaritonic states and for the THz mode as well as external quantum efficiencies for three types of THz emitters solving a set of corresponding equations for each case, i.e. Eqs. 4–7, 8–10 and 11–13, respectively. Consequently, Fig. 2 shows occupancies of the lower (a) and the upper (b) polariton states, occupancy of the THz mode (c) and external quantum efficiency for the emitter of type (i) using Eq. 2. This emitter utilizes the transition from the mixed state of the upper polariton and the dark exciton with the population *U* to the lower polariton with the population *L*. Dashed white lines in Fig. 2 represent isolines indicating that the total number of polaritons in the system is equal to 5 × 10^6^, corresponding to the excitonic Mott transition.

**Figure 3:**
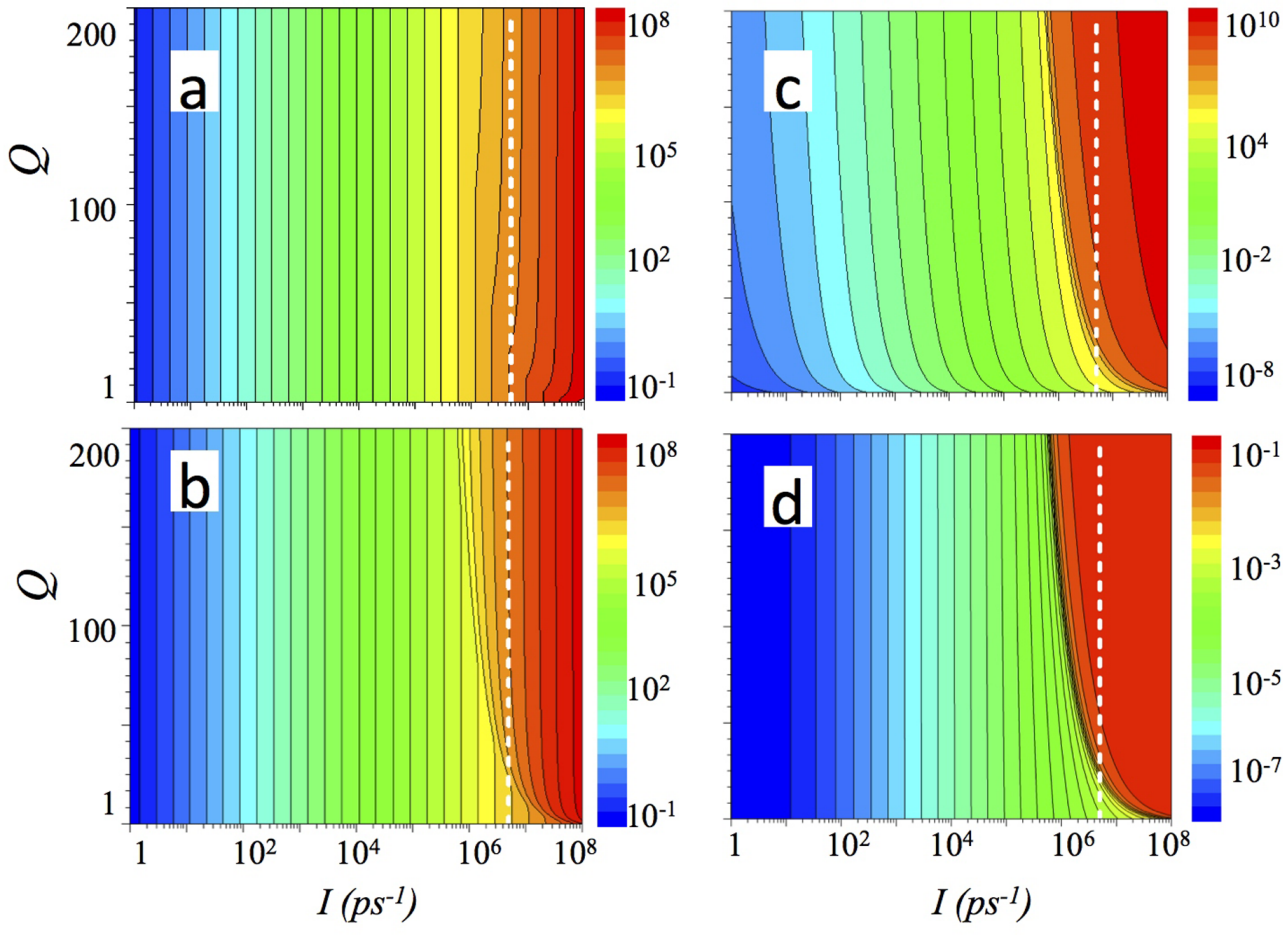
Populations (a) of the lower (L) and (b) of the upper (U) polaritonic levels, (c) of the THz mode T and (d) an external quantum efficiency as a function of pumping rate *I* and the quality factor of the THz cavity Q for the same polaritonic THz emitters of type (i) calculated using Eqs. 4–7. Here, Eq. 3 has been used for the emission probability. The following values were used for other parameters: *τ_L_* = 1 ps, *τ_U_* = 1 ps, *τ_R_* = 100 ps, *τ_UR_* = 10 ps, *τ_LR_* = 10 ps. The dashed white isoline shows a pumping level corresponding to 5 × 10^6^ polaritons in the system.

Similarly, Fig. 3 shows occupancies of the lower (a) and the upper (b) polariton states, population of the THz mode (c) and an external quantum efficiency (d) for the same emitter of type (i), calculated now using Eq. 3 for the emission probability.

**Figure 4:**
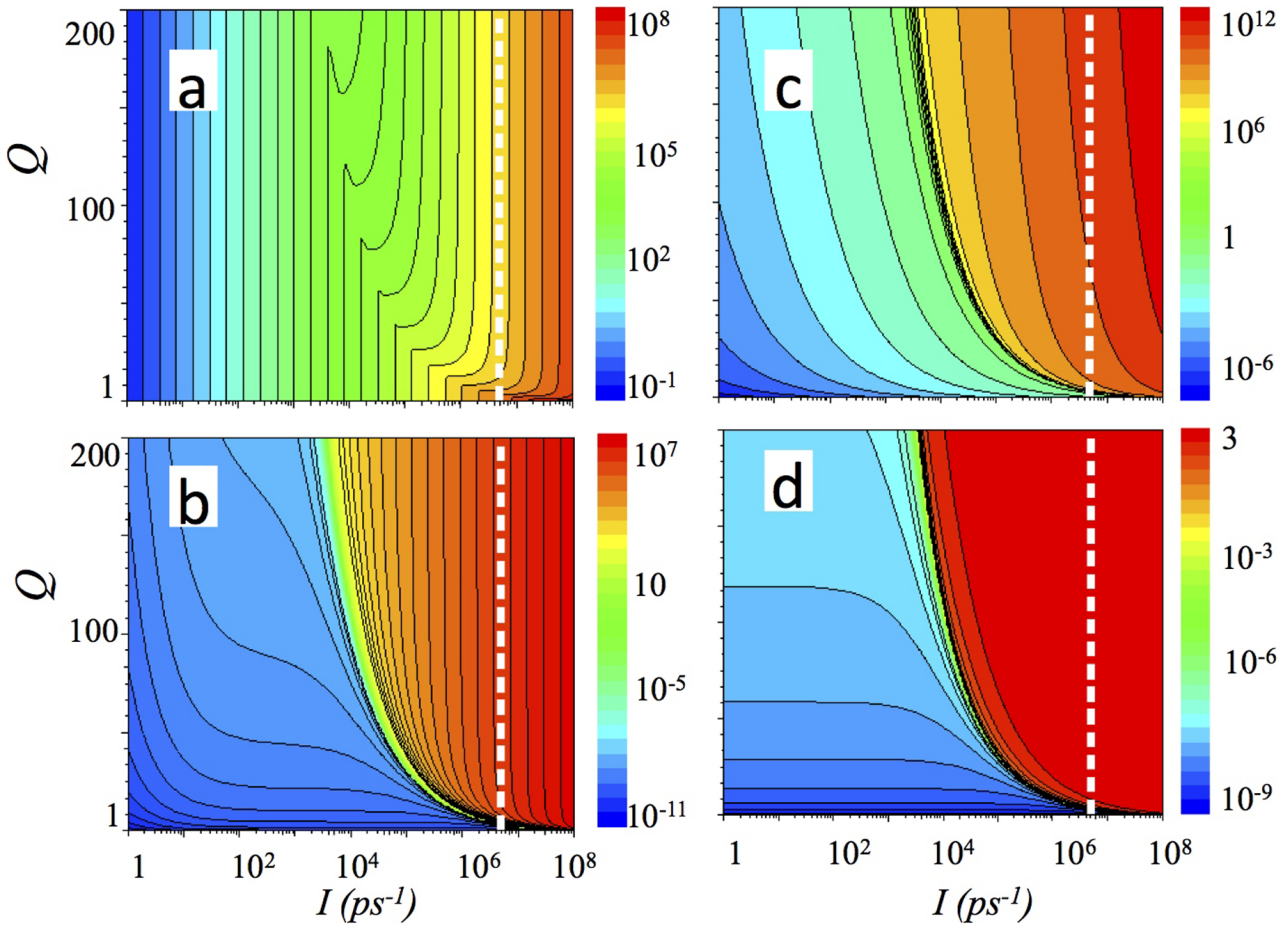
Populations of the highest (a) and the lowest (b) polariton level, THz mode (c), and an external quantum efficiency as a function of pumping rate *I* and quality factor of THz cavity Q for cascade polaritonic THz emitter described with 7 polaritonic levels^11^,^12^. A modelling was made using Eqs. 8–10 and Eq. 2 for emission probability. Parameters used for calculations: *τ_L_* = 1 ps, *τ_U_* = 1 ps, *τ_R_* = 100 ps, *τ_R+_* = 10 ps, *τ_R−_* = 10 ps, *τ_LR_* = 10 ps. The dashed white isoline shows a pumping level corresponding to 5 × 10^6^ polaritons in the system.

**Figure 5:**
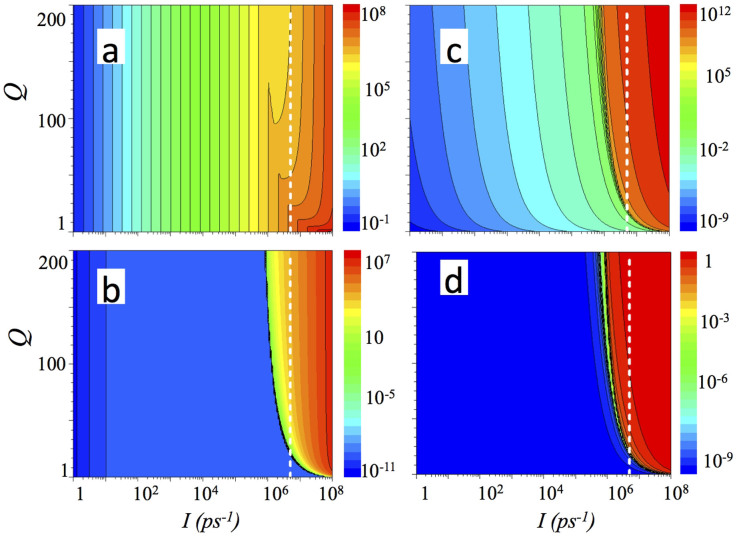
Populations of the highest (a) and the lowest (b) polariton level in the cascade, THz mode (c), and an external quantum efficiency as a function of pumping rate *I* and quality factor of THz cavity Q. Calculations are done using Eqs. 8–10 for the same cascade polaritonic THz emitter as in Fig.4, using now Eq. 3 for emission probability. Following parameters were utilized for modeling: *τ_L_* = 1 ps, *τ_U_* = 1 ps, *τ_R_* = 100 ps, *τ_R+_* = 10 ps, *τ_R−_* = 10 ps, *τ_LR_* = 10 ps. The dashed white isoline shows a pumping level corresponding to 5 × 10^6^ polaritons in the system.

**Figure 6:**
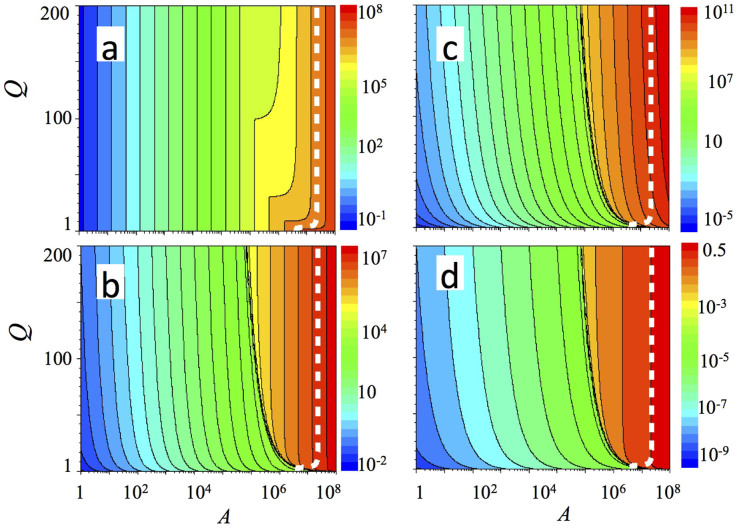
Occupations of 2*p*-exciton state (a) and *s*-polariton state (b), THz mode (c), and an external quantum efficiency (d) as a function of the occupancy of the pumping mode *A* and the quality factor Q for the THz cavity in the vertical cavity polaritonic THz emitters. Calculations are performed according to Eqs. 11–13. Parameters used are following: W_G_ = 10^−9^ ps^−1^, W_g_ = 10^−10^ ps^−1^, *τ_P_* = 100 ps, and *τ_S_* = 1 ps.

For the emitters based on polaritonic cascade lasers, the results are shown in Fig. 4 and Fig. 5 using Eq. 2 and Eq.3 for the emission probability, respectively. And, finally, we present results calculated for the type (iii) THz emitter, which is a vertical cavity polariton system utilizing transition between 2*p*-exciton and 1*s*-polariton. Fig. 6 (a), (b), (c) and (d) present occupancies (*P*) of 2p exciton, 1s –polariton (*S*), THz mode, and the quantum efficiency, respectively, for a vertical cavity polaritonic THz emitter.

## Discussion

It can be seen in Fig. 2, that the dependence of occupancy as a function of pumping rate *I* demonstrates a threshold–like behaviour, and that pumping above the threshold results in a saturation of the quantum efficiency at the level of ~0.5 (see Fig. 2(d)). Notably, this value is much higher than the efficiency of the quantum cascade laser^3^. When the pumping rate exceeds a critical value, marked by the isoline, indicating the total number of polaritons in the system corresponding to the Mott transition, excitons are screened and, thus, polaritonic effects, including radiative THz transitions, disappear. It can be seen that the minimal value of the quality factor Q required to achieve the threshold without the destruction of polaritons due to the excitonic Mott transition does not exceed 10. That is an encouraging result, since the Purcell factor in the considered system cannot exceed the ratio between the frequency of the emission and a width of the polaritonic level: when the energy level of an emitter is broadened, the cavity Purcell factor is replaced by the material Purcell factor, and even for ZnO based cavities it cannot exceed the level of few hundreds.

When the efficiency of the emission does not increase with the *Q*-factor of the cavity, lasing threshold occurs at a higher pumping level, but qualitatively the behaviour of the systems, described by equations (2) and (3), is similar.

The dependencies on pumping rate *I* and the quality factor *Q* calculated of the occupancy of the highest (with occupancy *P*_N_) and the lowest level (with occupancy *P*_0_) in the cascade (Fig. 4 (a,b)), using Eq. 2, are similar to the case of the emitter with a single THz transition shown in Fig. 2; however, the threshold is achieved at a slightly lower level of pumping intensity and sufficiently below the pumping level corresponding to the excitonic Mott transition. At the same time, the occupancy of the THz mode in Fig. 4(c) is several times larger and the quantum efficiency (Fig. 4(d)) saturates at the level exceeding unity. An interesting feature of the considered system is a non-linear, N-shaped dependence of the occupancy of the highest polariton level with pumping rate. Although S-shaped behaviour of the occupancy of the polariton modes due to a blue shift of the polariton level has already been experimentally demonstrated^27^, N–shaped behaviour opens new possibilities for applications of the polaritons in optical signal processing.

If the probability of spontaneous emission does not depend on the Q-factor, then, according to Eq. 3, the lasing threshold can be achieved at the higher pumping level, and in this case the external quantum efficiency has a smaller maximal value, as clearly shown in Fig. 5 (d).

We compare now results calculated for the third case (see Fig. 6), i.e. for a vertical cavity polariton emitter exploiting transition between 2*p*-exciton and 1*s*-polariton. In this case, the THz radiative emission corresponds to the transition from 2*p* exciton state to a lower polariton formed by 1 *s* exciton, while the pump of 2*p* exciton state is provided by two-photon absorption from “pumping” mode with occupancy *A*. Recently, such polaritonic emitter with a cavity for THz radiation has been theoretically considered^28^; however, no systematic analysis of the influence of the Q-factor of the THz cavity on the threshold pumping value was provided.

It can be seen that when the cavity for the THz radiation is absent (i.e. when *Q* = 1), the lasing threshold cannot be achieved for the polariton densities below the critical density of the excitonic Mott transition. An increase of *Q*-factor leads to the decrease of the threshold pumping, but the quantum efficiency cannot reach saturation, and it does not exceed 10^−2^ at the pumping level corresponding to the excitonic Mott transition. We note also, that the modelling carried out using Eqs. 11–13 does not consider the interaction of the polaritons with a reservoir (i.e. scattering of excitons and polaritons), which usually increases the threshold pumping and decreases the quantum efficiency.

Limiting mechanism preventing the achievement of the THz lasing threshold is the excitonic Mott transition. However, this effect is not investigated in detail. Various theoretical estimates suggest that the critical 2D excitonic Mott density corresponds to ~10^11^–10^12^ cm^−2^ 9,25. At the same time, experimental studies^29^, indicate that the Mott transition occurs gradually within the interval of the exciton density from ~10^10^ cm^−2^ to ~10^11^ cm^−2^. Thus, the value of critical concentration shown in figures 2–6 should be considered as an estimate, and can, in principle, vary within one order of magnitude in realistic structures.

In conclusion, we have analysed the influence of the cavity for THz radiation on polaritonic THz emitters. We have used two theoretical models: (i) when spontaneous emission rate of polaritons is proportional to the cavity Q-factor, and (ii) when the rate of emission is fixed. We have shown that in such polaritonic systems, where the polariton density required for the lasing threshold is above the critical density of the excitonic Mott transition, the THz lasing threshold cannot be achieved without an additional THz cavity. At the same time, embedding polaritonic microcavity into a cavity for the THz radiation even with a moderate Q-factor (up to 50) provide a THz lasing at pumping levels corresponding to the polariton densities below the excitonic Mott transition.

## Methods

We have done calculations of the population of polaritonic states and a THz mode for three different models of polaritonic THz emitters: (i) based on mixing of the upper polariton state with the dark exciton; (ii) based on polaritonic cascade lasers; and (iii) based on the transition between a dark 2*p*-exciton and a polariton condensate formed by an *s*-excitons.

For each system we analyse the population of polaritonic states and a THz mode using a set of steady-state Boltzmann equations, specific for each type of emitter, described in Ref. [8, 12,13,14].

The emitter of type (i) utilizes the transition from the mixed state of the upper polariton and the dark exciton with the population *U* to the lower polariton with the population *L*.

In an equilibrium state, it is described by a set of Boltzmann equations: 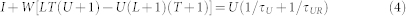

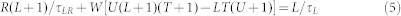


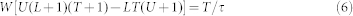

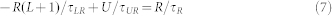
where R and T are occupancies of polaritonic reservoir and of THz mode, respectively; *τ_U_*, *τ_L_*, *τ_R_* and τ are the lifetimes of the upper polariton, lower polariton, polaritons in reservoir, and THz mode, respectively; 1*/τ_UR_* and 1*/τ_LR_* are the rate of polariton transition between the reservoir and the polaritonic upper and lower levels. The parameters used for modelling are the same as in Ref.(8) except for the values of the polariton lifetimes: in the current modelling we use, instead of the highest value corresponding to a fully optimized structure, the realistic value for a standard structure, which is equal to 1 ps.

For the emitters of type (ii) based on polaritonic cascade^12^,^13^, the occupancies in equilibrium can be described by the following equations: 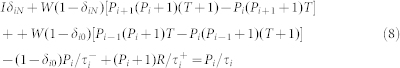

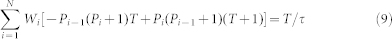

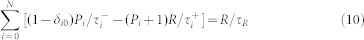
where *I* is the pumping intensity, *T*, *R* and *P_i_* (where *i* changes from 0 to *N*) –are occupancy numbers of the THz mode, polaritonic reservoir and polariton states, respectively; *W* is a probability of the radiative THz transitions between the polaritonic levels; 1/*τ_i_*^−^ and 1/*τ_i_*^+^ are the probabilities of the transitions from the polaritonic level with index “*i*” to the reservoir and back, respectively; *τ_R_*, 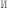 *τ_i_* are the lifetimes of polaritons in the reservoir and in the levels of the cascade, respectively.

Finally, as type (iii) emitter we consider a vertical cavity polariton emitter utilizing transition between 2*p*-exciton and 1*s*-polariton^14^. In the system of this type, the THz radiative transition occurs from 2*p* exciton state to a lower polariton formed by 1 *s* exciton, and the pumping of 2*p* exciton state is provided by a two-photon absorption from “pumping” mode (with an occupancy A). Occupancies of the *s*-exciton (*S*) and 2*p*-polariton (*P*) states in this situation are described by a set of equations: 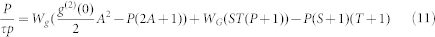

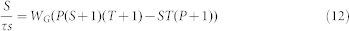

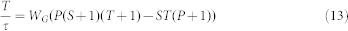
where *τ_p_* and *τ_s_* are the lifetimes of 2*p*-exciton and *s*- polariton, *W_g_* characterizes the probability of a two-photon absorption, and *g*^(2)^ is the second order coherence function.

For THz emitters of each type we have also calculated an external quantum efficiency according to *β = T/(τI)*, where *I* is a pumping intensity, *T* and τ is an occupancy and lifetime of corresponding THz mode, respectively.

## Author Contributions

M.A.K. designed the research idea. K.A.I. contributed to calculations. M.A.K., G.P. and A.J.G. contributed to discussion and writing the paper. All authors reviewed the manuscript.

## Figures and Tables

**Table 1:** Typical values of the Rabi splitting 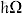 in bulk microcavities and in microcavities with embedded QWs, exciton Bohr radius *a*_B_, and dielectric constant *ε* together with the calculated maximal probability of the radiative transition for polaritons in bulk microcavity and in microcavities with QW based on GaAs, CdTe, GaN and ZnO

	GaAs, bulk	GaAs, QW	CdTe, bulk	CdTe, QW	GaN, bulk	GaN, QW	ZnO, bulk
Ω, _meV_	3	5–15	7	10–20	31	60	70–120
a_*B*_, nm	11.2	7	7	2.8	2.8	1.7	1.8
*ε*	13.1	n/a	10.4	n/a	9.5	n/a	7.7
W_max_, ms^−1^	2	7	7	10	80	150	250
